# Prioritizing functional modules mediating genetic perturbations and their phenotypic effects: a global strategy

**DOI:** 10.1186/gb-2008-9-12-r174

**Published:** 2008-12-16

**Authors:** Li Wang, Fengzhu Sun, Ting Chen

**Affiliations:** 1 Molecular and Computational Biology, Department of Biology Sciences, University of Southern California, 1050 Childs Way, Los Angeles, CA 90089-2910, USA; 2 MOE Key Laboratory of Bioinformatics and Bioinformatics Division, TNLIST/Department of Automation, Tsinghua University, Beijing 100084, PR China

## Abstract

We have developed a global strategy based on the Bayesian network framework to prioritize the functional modules mediating genetic perturbations and their phenotypic effects among a set of overlapping candidate modules. We take lethality in *Saccharomyces cerevisiae *and human cancer as two examples to show the effectiveness of this approach. We discovered that lethality is more conserved at the module level than at the gene level and we identified several potentially 'new' cancer-related biological processes.

## Abstract 2

A strategy is presented to prioritize the functional modules that mediate genetic perturbations and their phenotypic effects among candidate modules.

## Background

How to interpret the nature of biological processes, which, when perturbed, cause certain phenotypes, such as human disease, is a major challenge. The completion of sequencing of many model organisms has made 'reverse genetic approaches' [1] efficient and comprehensive ways to identify causal genes for a given phenotype under investigation. For instance, genome-wide knockout strains are now available for *Saccharomyces cerevisiae *[2,3], and diverse high throughput RNA interference knockdown experiments have been performed, or are under development, for higher organisms, including *C. elegans *[4], *D. melanogaster *[5] and mammals [6,7].

Compared to the direct genotype-phenotype correlation observed in the above experiments, what is less obvious is how genetic perturbation leads to the change of phenotypes in the complex of biological systems. That is, we might perceive the cell or organism as a dynamic system composed of interacting functional modules that are defined as discrete entities whose functions are separable from those of other modules [8]. For example, protein complexes and pathways are two types of functional modules. Using this concept as a basis for hypothesis, it is tempting to conclude that it is the perturbation of individual genes that leads to the perturbation of certain functional modules and that this, in turn, causes the observed phenotype. Previous studies have reported this type of module-based interpretation of phenotypic effects [9-11]. For example, Hart and colleagues [12] showed the distribution of gene essentiality among protein complexes in *S. cerevisiae *and suggested that essentiality is the product of protein complexes rather than individual genes. Other studies have made use of the modular nature of phenotypes to predict unknown causal genes [13]. In a recent study, Lage and colleagues [14] mapped diverse human diseases to their corresponding protein complexes and used such mapping to prioritize unknown disease genes within linkage intervals of association studies.

Despite these successful studies, the task of computationally inferring the functional modules that mediate genetic perturbations and their phenotypic effects might not be as easy as it appears. On the one hand, different modules could share common components. On the other hand, modules are believed to be hierarchically organized in biological systems [15] such that smaller modules combine to form larger modules, as shown in Gene Ontology (GO) annotations [16]. All these overlapping structures among modules make it difficult to accurately identify causal modules, the term we will use in this paper to indicate functional modules that mediate genetic perturbations and their phenotypic effects. To be more specific, since the protein products of a single gene could be associated with multiple modules, the phenotypic effects observed by perturbation of that gene could be attributed to the perturbation of any one of these modules, or their subsets. In other words, some modules, which are otherwise independent of a phenotype, but share members with actual causal modules of the phenotype, could be mistakenly prioritized as causal modules when traditional strategies, such as the hypergeometric (HG) enrichment test, are applied. This results from the fact that HG associates a module to the phenotype based merely on the phenotypic effects of its own components. In this paper, we refer to methods with the above characteristics as local strategies. We are therefore motivated to develop a global strategy, specifically, a Bayesian network (BN) model [17], to distinguish modules that are most likely to be actual causal modules from the other overlapping modules that are likely to be independent of the phenotype. We refer to this strategy as global since, in contrast to local strategy, it associates a module with a given phenotype based not only on its own components, but also on its overlapping structure with other modules. We applied the BN model to prioritize casual modules for two phenotypes: lethality in *S. cerevisiae *and human cancer. In both cases, as summarized below, we provide evidence indicating that the causal modules prioritized by the BN model are more accurate than those prioritized by such local strategies as the HG enrichment test. With lethality and human cancers as two illustrating examples, we aim to provide a general framework for module-based decoding of phenotypic variation caused by genetic perturbation, which could be applied to the understanding of diverse phenotypes in various organisms.

In the first case, we used gene lethality data observed from a genome-wide gene deletion study in *S. cerevisiae *[2]. Using the BN model, we then prioritized causal modules for which perturbation is the underlying cause of the inviable phenotype observed. For simplicity, we termed them as lethal modules, that is, lethal protein complexes or lethal biological processes. First, analysis of lethality of ortholog genes indicates that the BN model is superior to the HG enrichment test in distinguishing lethal protein complexes from non-lethal protein complexes. Moreover, in the course of the above analysis, we found that lethality is more conserved at the module level than at the gene level. Second, the module lethality inferred from the BN model is superior to the results obtained by the local strategy in predicting unknown lethal genes as evaluated through cross-validation.

In the second case, we applied our strategy to the study of human cancer. Human cancer is believed to be caused by the accumulation of mutations in cancer genes, for example, oncogenes and tumor suppressor genes. It has been suggested that a limited number of biological pathways might include most cancer genes [18]. Based on cancer genes documented in 'cancer-gene census' [19], we prioritized GO biological processes (BPs) causally implicated in cancers (CAN-processes). First, as indicated by their positions in the GO hierarchical structure and the conditional HG enrichment test, those GO BP nodes prioritized by the BN model are more likely to represent actual CAN-processes than those obtained by the HG enrichment test. Second, the results obtained from implementing the BN model are more consistent with previous knowledge of cancer-related processes than results obtained through the HG enrichment test. Third, similar to the case of lethality, the CAN-processes inferred from the BN model are superior to the results obtained by the local strategy in predicting unknown cancer genes as evaluated by cross-validation. Forth, by comparing the CAN-processes prioritized in 'cancer-gene census' to a recent set of cancer genes identified through systematic sequencing [20], we show that the results of our BN model, in contrast to the conditional HG enrichment test, are more consistent, even when different datasets of cancer genes are used. We also discuss the reasons that plausibly underlie the discrepancy between the results from the two datasets and identify and describe several potentially 'new' CAN-processes identified in the recent set of cancer genes, specifically, cytoskeleton anchoring and lipid transport.

## Results and discussion

### Prioritizing lethal modules in *S. cerevisiae*

We prioritized lethal modules from the gene lethality data in *S. cerevisiae *obtained from a genome-wide gene deletion study [2] (see Materials and methods). We provide evidence from two aspects indicating that the lethal modules prioritized by the BN model are more accurate than those prioritized by either the HG enrichment test or the local Bayesian (LM) model.

#### Superiority of the BN model indicated by analysis of lethality of ortholog genes

Compared with the HG enrichment test, our analysis of lethality of ortholog genes in the context of protein complexes indicates that the BN model is superior in distinguishing lethal from non-lethal protein complexes. It is difficult to directly measure the accuracy of the prioritized lethal protein complexes without a direct benchmark for lethal and non-lethal protein complexes. However, we expect that genes involved in lethal protein complexes will show some characteristics that distinguish them from genes that do not possess such characteristics. These characteristics could therefore serve as indicators of lethality of protein complexes and, hence, could be used to measure the quality of the prioritized data. Here, we consider one such potential characteristic, as described below.

We can categorize non-lethal genes into two classes according to the lethality of protein complexes in which they participate. For simplicity, we refer to non-lethal genes whose protein products have been involved in certain lethal protein complexes as NLGLCs, and we refer to non-lethal genes whose protein products have not been involved in any lethal protein complexes as NLGNLCs. A key computational measurement we use is termed 'ortholog lethal ratio,' which refers to the proportion of genes in species A, specifically *S. cerevisiae*, whose ortholog genes in species B, specifically *C. elegans*, are lethal. Thus, we hypothesize that NLGLC has a higher 'ortholog lethal ratio' than NLGNLC. An intuitive argument supporting this theory is that, in order for those NLGNLCs in *S. cerevisiae *to evolve into lethal genes in *C. elegans*, they must undergo some extra evolutionary events that associate their protein products with certain lethal modules, which would be a prerequisite for genes showing inviable phenotype when perturbed under a module-based explanation of lethality. On the other hand, since NLGLCs by definition already meet this requirement, and assuming module lethality and composition are relatively conserved across species, it might be easier for them to evolve into lethal genes in *C. elegans*, for instance, by losing their functional backup within lethal modules. Here, we only focus on non-lethal genes, either NLGLC or NLGNLC, but not lethal genes because, according to the module-based explanation of gene lethality, all lethal genes must have been involved in certain lethal modules, and there is no such classification in the case of non-lethal genes. Nevertheless, in the following analysis, we also categorized lethal genes into two classes in a manner similar to non-lethal genes, namely, lethal genes whose protein products have been involved in certain lethal protein complexes (referred to as LGLCs for simplicity) and lethal genes whose protein products have not been involved in any lethal protein complexes (referred to as LGNLCs for simplicity). It should be noted that such classification is simply for the purpose of elucidation. Not all lethal modules are included in our dataset. Thus, the existence of LGNLCs that have not been associated with any lethal modules in our dataset largely results from data incompleteness.

**Figure 1:**
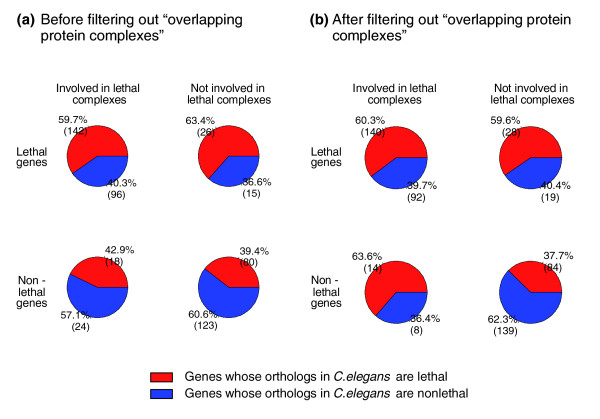
**Genes in *S. cerevisiae *are classified into four groups according to their lethality and the lethality of protein complexes to which they belong**. Within each group, the pie chart represents the distribution of genes with respect to the lethality of their orthologs in *C. elegans*. **(a) **The lethal protein complexes were identified using the HG enrichment test (*p*-value < 0.05). **(b) **'Overlapping protein complexes' (the probability of being lethal inferred by the BN model < 0.7) were filtered out from those identified in (a).

Since we are able to distinguish lethal from non-lethal protein complexes based on the 'ortholog lethal ratio' of their associated non-lethal genes, we could expect that a list of protein complexes with a higher enrichment of lethal protein complexes will show a higher 'ortholog lethal ratio' of non-lethal genes than otherwise. We therefore carried out the following analysis to compare the capacity of the HG enrichment test with the BN model in distinguishing lethal from non-lethal protein complexes. To determine the lethality of protein complexes, we first employed the HG enrichment test to evaluate the enrichment of lethal genes in 390 curated protein complexes in *S. cerevisiae*. More specifically, we assume each protein complex as a random sample from a set of 5,916 genes, 1,105 of which are lethal. We called a complex with Nc genes and Lc lethal genes lethal if the probability of having at least Lc lethal genes out of Nc genes is less than 0.05 based on the hypergeometric distribution. We obtained a total of 149 lethal protein complexes in this way. We then classified genes into four groups according to their gene lethality and the lethality of protein complexes in which they participate: LGLC, LGNLC, NLGLC and NLGNLC. To estimate the 'ortholog lethal ratio' for each group of genes, we calculated the proportion of genes whose orthologs in *C. elegans *are lethal among all the genes whose orthologs in *C. elegans *exist with known lethality (see Materials and methods for details of gene lethality data in *C. elegans*). As shown in Figure 1a, there appears to be no significant difference between NLGLCs and NLGNLCs derived in this way (lower left and right cells, respectively), as indicated by the 'ortholog lethal ratio' (*p*-value of chi-square test between the two groups > 0.1). However, as discussed in the Background, the above HG method might overestimate the number of lethal complexes by including 'overlapping protein complexes' whose enrichment of lethal genes would most likely result from the sharing of gene members with actual lethal protein complexes. Thus, we then used the BN model to filter out those 'overlapping protein complexes'. Out of the above 149 protein complexes with an HG *p*-value < 0.05, we filtered out 55 protein complexes whose probability of being lethal, as derived from the BN model, was < 0.7 and treated them as non-lethal protein complexes. In this case, the 'ortholog lethal ratio' is significantly higher for NLGLCs than for NLGNLCs after filtering out the 'overlapping protein complexes' (lower left and right cells, respectively, of Figure 1b; *p*-value of chi-square test between the two groups < 0.05). It has to be mentioned that those protein complexes that are not significantly enriched with lethal genes (*p*-value of HG enrichment test > 0.05) are not considered as candidate lethal protein complexes in the BN model to speed up the algorithm, since those HG insignificant complexes are of less practical use and could add a substantial amount of computational burden to the BN model, particularly when GO BPs are considered in later analysis. Other preprocessing strategies to speed up the algorithm might work as well, for instance, removing protein complexes with the number of lethal genes less than a threshold.

**Figure 2:**
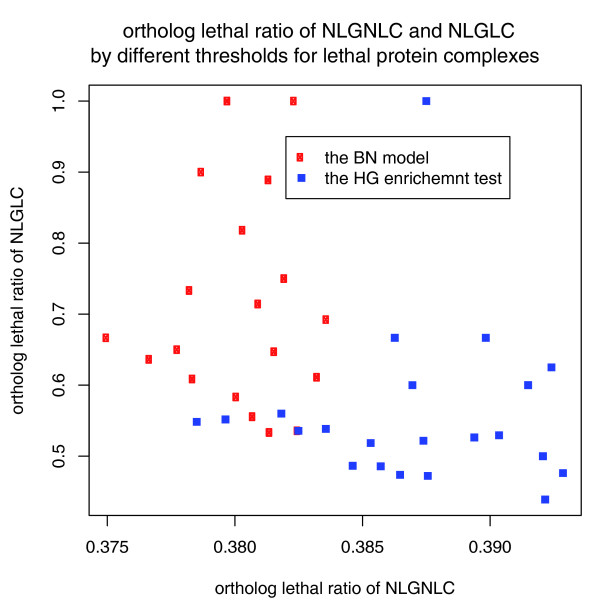
**The 'ortholog lethal ratio' for NLGLC and NLGNLC when a more stringent cutoff of *p*-value (<0.05) of the HG enrichment test is used to identify lethal protein complexes (blue), or a different cutoff of the probability of being lethal inferred by the BN model (red) is used to filter out 'overlapping protein complexes'**.

Based on the results of the above analysis, we conclude that the BN model is superior to the HG enrichment test in distinguishing lethal protein complexes from non-lethal protein complexes as indicated by the following four findings. First, as indicated by the 'ortholog lethal ratio,' those 'overlapping protein complexes' filtered out by the BN model are very likely to be non-lethal protein complexes. To be more specific, the 'ortholog lethal ratio' for non-lethal genes only involved in the 'overlapping protein complexes' was not found to be significantly different (20%) from that of NLGNLCs before filtering (39.4%; lower right cell in Figure 1a). However, it was found to be significantly lower than that of NLGLCs after filtering (63.6%; lower left cell in Figure 1b; *p*-value of chi-square test < 0.05). In other words, by successfully filtering out these 'overlapping protein complexes,' the resulting list of lethal protein complexes becomes more enriched when quantified by the 'ortholog lethal ratio'. Second, in the absence of the BN model, it is unlikely that those 'overlapping protein complexes' could have been effectively filtered out by the HG enrichment test, even by setting a more stringent *p*-value cutoff, since, based on the Wilcoxon rank-sum test, there is no significant difference between the HG *p*-value of those 'overlapping protein complexes' filtered out by the BN model and the HG *p*-value of the remaining lethal protein complexes. Third, the coverage of lethal genes by lethal protein complexes remains similar, both before and after filtering out 'overlapping protein complexes'. Because the 'overlapping protein complexes' filtered out by the BN model are those sharing lethal gene members with the remaining lethal protein complexes, it can be seen from the data in Figure 1 that the number of distinct lethal genes covered by lethal protein complexes after filtering (140 + 92; upper left cell in Figure 1b) is only marginally smaller than before filtering (142 + 96; upper left cell in Figure 1a). If, however, a more stringent cutoff *p*-value is set for the HG enrichment test, the coverage of lethal genes by lethal protein complexes will be dramatically decreased (data not shown). Fourth, even when the coverage of lethal genes is not considered, the BN model still performs better than the HG enrichment test in distinguishing lethal protein complexes from non-lethal protein complexes as measured by the 'ortholog lethal ratio' of non-lethal genes. Figure 2 shows the 'ortholog lethal ratio' for NLGLCs and NLGNLCs (lower left and right cells, respectively, in Figure 1a or 1b) when different thresholds for either the *p*-value of the HG enrichment test or the probability of being lethal protein complexes derived from the BN model are used. Compared to the HG enrichment test, it can clearly be seen that the 'ortholog lethal ratio' shows more striking differences between NLGLCs and NLGNLCs when the BN model is used.

The analysis of the lethality of ortholog genes in the context of protein complexes also reveals that lethality is more conserved at the module level than at the gene level. In other words, compared with the lethality of a gene itself, the lethality of the protein complexes in which that gene participates appears to be a more relevant predictor for the lethality of its orthologs in other organisms. It can be seen that both LGLCs and NLGLCs show a similar 'ortholog lethal ratio' (upper and lower left cells in Figure 1b), which is significantly higher than that of NLGNLCs (lower right cell in Figure 1b). It should be noted that a similar pattern could be observed when the 'ortholog lethal ratio' is calculated based on essential genes in *D. melanogaster *instead of *C. elegans *(Figure S1 in Additional data file 1). This indicates that our observations here are not restricted to one dataset or one species. Since the genome-wide whole organism screening is not available for *D. melanogaster*, the gene lethality in *D. melanogaster *is defined based on cell-based RNA interference screening [21]. The ortholog lethal ratio might be underestimated in this way because genes that are lethal to the whole organism might not display any phenotype when tested in certain types of cells. It may be recalled from our discussion above that LGNLCs (upper right cell in Figure 1b) may theoretically belong to some other lethal modules, thus showing a high 'ortholog lethal ratio' comparable to LGLCs.

Our finding that lethality is more conserved at the module level than at the gene level has several important implications. First, it could serve as a piece of evolutionary evidence supporting the modular nature of lethality. Second, to supplement traditional gene-based mapping, it suggests that a module-based mapping strategy might be employed in transferring phenotypic knowledge across species where it is the phenotypic effects of the associated modules, rather than the phenotypic effects of individual genes, that are believed to be conserved across species. For example, we want to predict ortholog lethality in *C. elegans *from lethality data in yeast. According to the traditional sequence-similarity mapping, the orthologs of LGLCs and NLGLCs are predicted as lethal and non-lethal, respectively. However, according to our analysis (Figure 1b), NLGLCs show a similar 'ortholog lethal ratio' to that of LGLCs. Thus, it might be useful to predict the orthologs of NLGLCs as lethal instead of non-lethal. By doing so, more lethal genes can be predicted, but the accuracy (defined as the fraction of true lethal genes among all the predicted lethal genes) remains similar, which is around 60% in the case of *C. elegans*.

Analysis of the proportion of lethal genes in each of the 94 curated lethal protein complexes identified by the BN model reveals a high modularity of lethality. As shown in Table S1 in Additional data file 1, all the members of about 63.8% (60 out of 94) of them are lethal; more than half of the members are lethal in all except for one of them. In addition, the proportion of lethal genes in a lethal complex appears to differ based on their functions. For example, as listed in Table S1, lethal complexes related to chromatin remodeling, such as the RSC complex and the INO80 complex, or protein transport and translocation, such as the mitochondrial outer membrane translocase complex, nuclear pore complex, and ER protein-translocation complex, have a relatively low proportion of lethal genes. The relatively low proportion of lethal genes indicates functional redundancy within those complexes. For example, the nuclear pore complex has the principal function of regulating the high throughput of nucleocytoplasmic transport in a highly selective manner [22]. The fact that over half the total mass of FG domains could be deleted without loss of viability or the nuclear pore complexe's normal permeability barrier suggests the existence of multiple translocation pathways and partial redundancy among them [23].

#### Superiority of the BN model revealed by cross-validation

Besides the above ortholog lethality analysis, we also compared the power of the BN model with the local strategy in predicting unknown lethal genes. The module lethality inferred from the BN model is superior to the results obtained by the local strategy in predicting unknown lethal genes as evaluated by cross-validation. As mentioned before, one of the applications of identifying causal modules is the prediction of unknown causal genes. However, for gene lethality in *S. cerevisiae*, this is not necessary since the lethality of almost all the genes is known. Nonetheless, *S. cerevisiae *does provide a good system for evaluating prediction accuracy of gene lethality through cross-validation. In the context of our study, if, by such evaluation, we assume that more accurate prediction of gene lethality is a consequence of more accurate inference of module lethality, then prediction accuracy of the former could reflect prediction accuracy of the latter.

**Figure 3:**
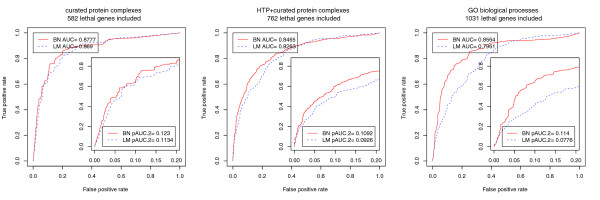
**The ROC curve, AUC and pAUC.2 of 100-fold cross-validation in predicting lethality of genes in *S. cerevisiae *using (a) curated protein complexes, (b) curated and HTP protein complexes and (c) GO biological process**. BN represents the BN model, and LM represents the local Bayesian model.

To evaluate prediction accuracy of gene lethality through cross-validation, we randomly chose part of the gene lethality data (training data) as a known to estimate module lethality. The estimation results were then used to infer the probability of being lethal for the remaining genes (testing data; see Materials and methods for details). In the step where the lethality of each candidate module is inferred, we employed the BN model as our global strategy and the LM model as our local strategy with the purpose of comparing how the results of these two methods could affect prediction accuracy of gene lethality. The LM model differs from the BN model only in that only the subnetwork for a candidate module is considered as if none of its components participates in other modules (see Materials and methods for details). In this sense, the probability of being lethal for each protein complex inferred by the LM model is similar to the *p*-value of the HG enrichment test in prioritizing lethal protein complexes. In this case, we chose to compare the BN model with the LM model instead of the HG enrichment test. Compared with the *p*-value derived from the HG enrichment test, the output of the LM model is more like the BN model and, therefore, it is easier to infer gene lethality with it. We used the receiver operating characteristic (ROC) curve [24] and the area under the ROC curve (AUC) of 100-fold cross-validation as measurements of the prediction accuracy of unknown lethal genes. We calculated both standard AUC and partial AUC (pAUC) [25] at a false positive rate of 0.2 (denoted as pAUC.2). Because the BN model is primarily designed to remove potential false positives that are overestimated by the HG/LM method, we are predominantly concerned with the prediction accuracy of our models at low false positive rates [26], which are preferred in practice. The results are shown in Figure 3.

When the candidate modules consist of only curated protein complexes, the pAUC.2 of our BN model increases by 8.5% compared with that of the LM (Figure 3a). The relatively smaller improvement in this case might be a result of the fact that the AUC is already very high with curated protein complex data. As a matter of fact, when the HTP protein complex data are added to the candidate modules, the pAUC.2 increases by 17.9%, which is more visible (Figure 3b). The pAUC.2 increases by 46.9% when GO BPs are considered as candidate modules (Figure 3c). Since the BN model is designed to accommodate the overlapping structures among functional modules, such a striking improvement is consistent with the more complicated overlapping structures among GO BPs. Our simulation results (Additional data file 1) also show that the amount of improvement of the BN model over the HG method in identifying causal modules increases as the degree of overlap among modules increases (Figure S2 in Additional data file 1). Since both methods perform similarly at high false positive rates, the average improvement over the whole range of false positive rates is relatively small. The standard AUC of the BN model increases by 1%, 2.4% and 7.6% for the three cases (Figure 3abc), respectively. Therefore, our results show that the module lethality inferred by the BN model is superior to the results obtained by the LM model in predicting unknown lethal genes. Overall, therefore, to the extent that prediction accuracy of gene lethality reflects prediction accuracy of module lethality, our results also indicate that the lethal modules identified by the BN model are more accurate than those identified by the local strategy.

### Prioritizing GO biological processes causally implicated in human cancer

In order to show how the BN model could be applied to more complicated phenotypes, such as human diseases, we prioritized GO BPs that are causally implicated in human cancers (CAN-processes) based on cancer genes documented in 'cancer-gene census', a curated cancer gene database assembled from previous studies [19]. Compared with protein complexes, BPs are more conceptually defined modules whose interrelationships appear to be more complicated. For example, the GO BPs [16] are organized into a directed acyclic structure, where children nodes representing BPs with more specific definition are pointed into parent nodes representing BPs with broader definition. Such a hierarchical organization makes it possible to investigate the biological system with varied specificity, but also brings in some difficulties. For example, if one GO BP node is enriched for lethal genes based on the HG enrichment test, it is very likely that many of its offspring nodes and ancestor nodes are also enriched, as well as some nodes that share members with it. However, since our BN model is a global strategy sensitive to the interrelationship among modules, it might be more useful than the HG enrichment test (local strategy) in distinguishing GO BP nodes that are most likely to represent actual CAN-processes from those whose enrichment of cancer genes is more peripheral, either from sharing members with them or being their ancestor or offspring nodes. For simplicity, we refer to the latter as 'overlapping GO BP nodes'. Using measurement parameters similar to those of our gene lethality model, only GO BP nodes with a HG enrichment test *p*-value < 0.05 are treated as candidate modules in the BN model, and the same empirical cutoff was used to filter out 'overlapping GO BP nodes'. Table 1 lists the resulting GO BP nodes and the same number of GO BP nodes prioritized by the HG enrichment test. Our results show that the GO BP nodes identified by the BN model are likely to be better representatives of CAN-processes than those identified by the HG enrichment test in three different respects.

**Table 1:** The 27 GO CAN-processes prioritized by the BN model or the HG enrichment test based on cancer genes from the 'cancer-gene census' database

GO CAN-processes prioritized by the BN model			GO CAN-processes prioritized by the HG enrichment test		
	
GO CAN-process	Total gene number	Cancer gene number	GO CAN-process	Total gene number	Cancer gene number
GO:0006366 transcription from RNA polymerase II promoter	541	52	GO:0050794 regulation of cellular process	3,958	205
GO:0045737 positive regulation of cyclin-dependent protein kinase activity	3	3	GO:0050789 regulation of biological process	4,256	209
GO:0045786 negative regulation of progression through cell cycle	203	41	GO:0065007 biological regulation	4,648	217
GO:0007169 transmembrane receptor protein tyrosine kinase signaling pathway	168	23	GO:0043283 biopolymer metabolic process	5,095	226
GO:0048268 clathrin cage assembly	4	2	GO:0000074 regulation of progression through cell cycle	325	53
GO:0000718 nucleotide-excision repair, DNA damage removal	21	7	GO:0051726 regulation of cell cycle	329	53
GO:0002903 negative regulation of B cell apoptosis	2	2	GO:0019219 regulation of nucleobase, nucleoside, nucleotide and nucleic acid metabolic process	2,501	145
GO:0015014 heparan sulfate proteoglycan biosynthetic process, polysaccharide chain biosynthetic process	3	2	GO:0031323 regulation of cellular metabolic process	2,703	151
GO:0010225 response to UV-C	2	2	GO:0006350 transcription	2,540	145
GO:0006310 DNA recombination	92	13	GO:0019222 regulation of metabolic process	2,832	154
GO:0016571 histone methylation	6	2	GO:0006139 nucleobase, nucleoside, nucleotide and nucleic acid metabolic process	3,771	181
GO:0060070 Wnt receptor signaling pathway through beta-catenin	5	2	GO:0045449 regulation of transcription	2,448	140
GO:0016573 histone acetylation	10	4	GO:0006351 transcription, DNA-dependent	2,360	136
GO:0045429 positive regulation of nitric oxide biosynthetic process	5	3	GO:0006355 regulation of transcription, DNA-dependent	2,302	134
GO:0006298 mismatch repair	31	7	GO:0045786 negative regulation of progression through cell cycle	203	41
GO:0009168 purine ribonucleoside monophosphate biosynthetic process	15	2	GO:0032774 RNA biosynthetic process	2,364	136
GO:0010332 response to gamma radiation	3	2	GO:0043170 macromolecule metabolic process	6,647	244
GO:0045661 regulation of myoblast differentiation	6	2	GO:0022402 cell cycle process	606	61
GO:0030101 natural killer cell activation	15	3	GO:0016070 RNA metabolic process	2,896	143
GO:0046902 regulation of mitochondrial membrane permeability	5	2	GO:0007049 cell cycle	761	67
GO:0051353 positive regulation of oxidoreductase activity	5	2	GO:0048523 negative regulation of cellular process	917	73
GO:0051898 negative regulation of protein kinase B signaling cascade	2	2	GO:0048519 negative regulation of biological process	958	73
GO:0000910 cytokinesis	28	4	GO:0044238 primary metabolic process	7,595	254
GO:0000075 cell cycle checkpoint	58	14	GO:0048522 positive regulation of cellular process	754	63
GO:0001952 regulation of cell-matrix adhesion	9	6	GO:0006366 transcription from RNA polymerase II promoter	541	52
GO:0042593 glucose homeostasis	11	2	GO:0048518 positive regulation of biological process	840	65
GO:0014065 phosphoinositide 3-kinase cascade	5	3	GO:0009719 response to endogenous stimulus	400	44
Median number	6	3	Median number	2,364	136

First, as indicated by their positions in the GO hierarchical structure and the conditional HG enrichment test, those GO BP nodes prioritized by the BN model are more likely to represent actual CAN-processes than those obtained by the HG enrichment test. We plotted the 27 BP nodes prioritized by the BN model (as listed in Table 1) together with all their offspring and ancestor nodes in the directed acyclic structure (Additional data file 2). It can be seen that most of the nodes in this subgraph are significantly enriched with cancer genes (The node size in Additional data file 2 corresponds to the minus log *p*-value of the HG enrichment test.) As noted above, if one GO node is enriched with cancer genes, many of its ancestor and offspring nodes will also become enriched. The results shown in Additional data file 2 are, therefore, consistent with this observation. It can also be seen that most GO BP nodes prioritized by the HG enrichment test (23 out of 27 GO BP nodes as listed in Table 1) are also within this subgraph. However, while most of the 27 GO BP nodes prioritized by the BN model are close to the leaf nodes, those prioritized by the HG enrichment test are close to the root.

Since most GO BP nodes prioritized by the HG enrichment test are close to the root node, it is suspected that the enrichment of cancer genes for most of them might actually result from being ancestor nodes of actual CAN-processes. As a matter of fact, the enrichment of cancer genes for 63.0% of these nodes (17 out of 27) becomes insignificant (*p*-value of the HG enrichment test > 0.05) conditional on at least one of its child nodes [27]. In order to calculate the *p*-value of the HG enrichment test of node A conditional on node B, we removed genes included in node B from node A and calculated the *p*-value of the enrichment of cancer genes for the remaining genes in node A. As a comparison, since the 27 GO BP nodes prioritized by the BN model are close to the leaf nodes, their enrichment of cancer genes is less likely to result from being ancestor nodes of actual CAN-processes. As a matter of fact, out of 16 nodes that are not leaf nodes, only 12.5% (2 out of 16) become insignificant conditional on at least one of their child nodes. Moreover, for the two nodes that become insignificant conditional on their child nodes, none of their child nodes is significantly enriched with cancer genes (*p*-value of the HG enrichment test > 0.05). In this sense, their child nodes are not better representatives of actual CAN-processes than the two nodes themselves.

**Figure 4:**
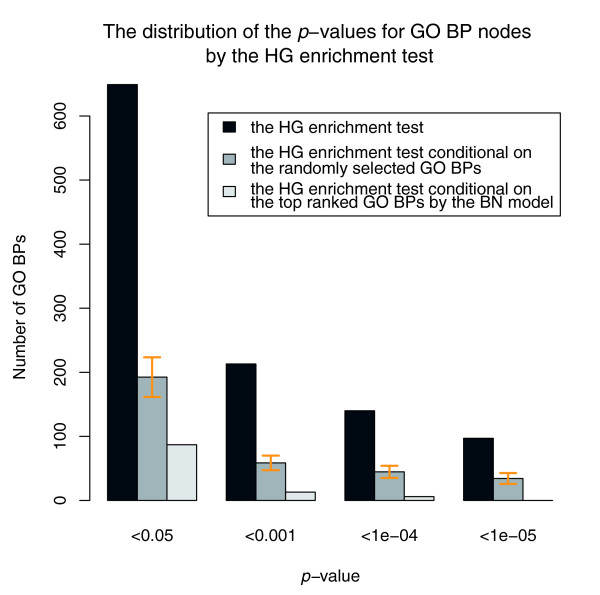
**The distribution of *p*-values for the enrichment of cancer genes for GO BP nodes, by the HG enrichment test, the HG enrichment test conditional on the 27 CAN-processes prioritized by the BN model, and the HG enrichment test conditional on the same number of randomly sampled GO BP nodes with similar size**. The error bars stand for the standard deviation of the corresponding quantities.

On the other hand, although most GO BP nodes prioritized by the BN model are of smaller size and close to the leaf nodes, their enrichment of cancer genes is less likely to result from being the offspring nodes of actual CAN-processes. This means that only a few of their ancestor nodes will remain significantly enriched conditional on the 27 GO BP nodes prioritized by the BN model. In order to demonstrate this, for each parent node of the 27 GO BP nodes prioritized by the BN model, we calculated the *p*-value of the HG enrichment test conditional on the 27 nodes. Only 6.8% (3 out of 44) of their parent nodes were conditionally significant (*p*-value < 0.05). We then extended such a conditional HG enrichment test to all 649 GO BP nodes that are enriched with cancer genes (*p*-value of the HG enrichment test < 0.05). The distribution of the original *p*-values of the HG enrichment test and the *p*-values of the HG enrichment test conditional on the 27 GO BP nodes are shown in Figure 4. It can be seen that most GO BP nodes become insignificant conditional on the 27 CAN-processes prioritized by the BN model (*p*-value > 0.05); only 13 have a *p*-value < 0.001 and none have a *p*-value < 1e-5. It can also be seen in Figure 4 that the number of significantly enriched GO BP nodes conditional on the 27 CAN-processes is significantly smaller than the number of significantly enriched GO BP nodes conditional on the same number of randomly selected GO BP nodes with similar size.

Second, the results obtained from implementing the BN model are more consistent with previous knowledge of cancer-related processes than the results obtained through HG enrichment test results. As shown in Table 1, a variety of well-known cancer-related processes have been prioritized by the BN model. They include those directly related to cell cycle - for example, positive regulation of cyclin-dependent protein kinase activity and cell cycle checkpoint - and those canonical signaling pathways regulating cell birth and death [18] - for example, the transmembrane receptor protein tyrosine kinase signaling pathway, the Wnt receptor signaling pathway through beta-catenin, the phosphoinositide 3-kinase cascade and the protein kinase B signaling cascade. They also include biological processes responsible for the maintenance of genome stability [28] - for example, nucleotide-excision repair, DNA damage removal and mismatch repair - or epigenetic modification [29] - for example, histone methylation and histone acetylation. The associations of some prioritized CAN-processes with cancers might be less apparent, but the literature has indicated their involvement with more well-known CAN-processes. For example, the role of clathrin cage assembly in cancer generation might be related to its function in controlling epidermal growth factor receptor signaling through clathrin-mediated endocytosis [30]. Another example is regulation of mitochondrial membrane permeability, whose role in apoptosis has been shown before [31]. On the other hand, the CAN-processes prioritized by the HG model might be too generally defined to be associated with cancers. As shown in Table 1, most of the CAN-processes prioritized by the HG enrichment test are >2,000 in size, which renders them less informative.

**Figure 5:**
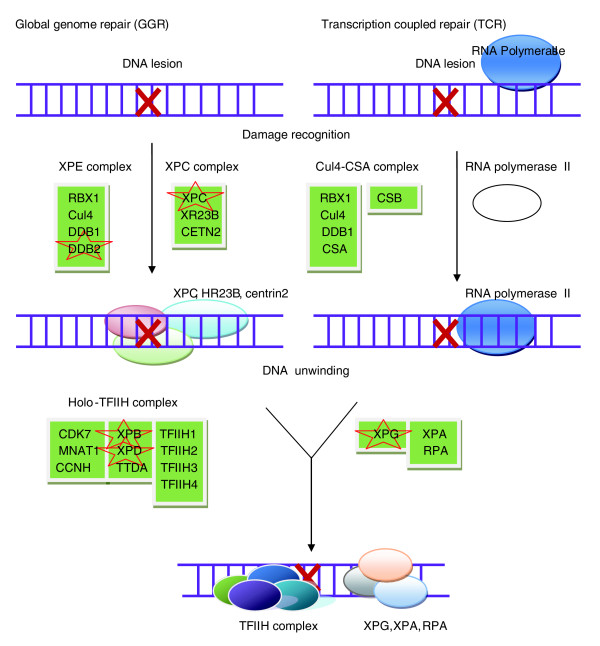
**Pathways of global genome nuclear excision repair (GG-NER) and transcription-coupled nuclear excision repair (TC-NER)**. Cancer genes involved in the two subpathways as documented in 'cancer gene census' are marked by red stars. In GG-NER, damage, such as ultraviolet-induced cyclobutane pyrimidine dimers (CPD) or 6-4 photoproducts (6-4 PP), is recognized by proteins, including the XPE (DDB2) and XPC gene products. In TC-NER, the lesion appears to block the progress of RNA polymerase II in a process involving the CSA and CSB gene products. Following initial damage recognition, the two subpathways converge. The XPB (ERCC3) and XPD (ERCC2) helicases unwind the region surrounding the lesion, along with the XPA and XPG (ERCC5) gene products and replication protein A (RPA). (The graph was obtained from the KEGG Pathway database [52], and only part of it is shown here.)

Previous knowledge also indicates that some of the 'overlapping GO BPs' filtered out by the BN model might be independent of cancer. Importantly, in the absence of such a global approach, these 'overlapping GO BPs' are less distinguishable from actual CAN-processes based on the HG enrichment test. One example is nuclear excision repair (NER), which can be categorized into two classes: global genome NER (GG-NER) and transcription coupled NER (TC-NER) [32]. The two subpathways differ in the sets of proteins involved in the distortion and recognition of the DNA damage, but converge after that (Figure 5). Out of a total of 21 genes involved in GG-NER based on GO annotations, 7 have been documented as cancer genes in 'cancer-gene census'. Similarly, three out of six genes involved in TC-NER have been documented as cancer genes. Based on the HG enrichment test, both GG-NER and TC-NER are significantly enriched with cancer genes, along with their parent node NER, with *p*-values of 2e-07, 2e-04 and 2e-06, respectively. However, under the BN model, only GG-NER was prioritized among the top list, while TC-NER and NER are filtered out as 'overlapping GO BPs'. When we take a close look at the exact position of those cancer genes in the two subpathways, it can be seen that all three cancer genes involved in TC-NER, that is, XPB (ERCC3), XPD (ERCC2) and XPG (ERCC5), function after the two subpathways converge. None of the genes involved in the initial damage recognition, which is specific to TC-NER, for example, CSA (ERCC8) and CSB (ERCC6), has yet been documented as a cancer gene in 'cancer-gene census'. On the other hand, a number of genes specific to GG-NER, for example, XPE (DDB2) and XPC, have been documented as cancer genes. Therefore, it is speculated that TC-NER itself might not be a CAN-process. Such a hypothesis has been supported by previous studies. For example, it has been shown that skin cancer is not a feature of pure Cockayne syndrome, a disease that could be caused by defects in gene CSA or CSB [33]. Since, as described above, both CSA and CSB are specific to TC-NER, such an observation indicates that pure perturbation of TC-NER might not cause cancer. A more comprehensive survey regarding the relationship between GG-NER and TC-NER can be found in [32]. Nevertheless, since our knowledge of cancer genes is far from complete, the case about the role of TC-NER in cancers remains to be elucidated. In this regard, it might be more precise to treat those 'overlapping modules' filtered out by the BN model as those cases where further investigation and justification are needed.

**Figure 6:**
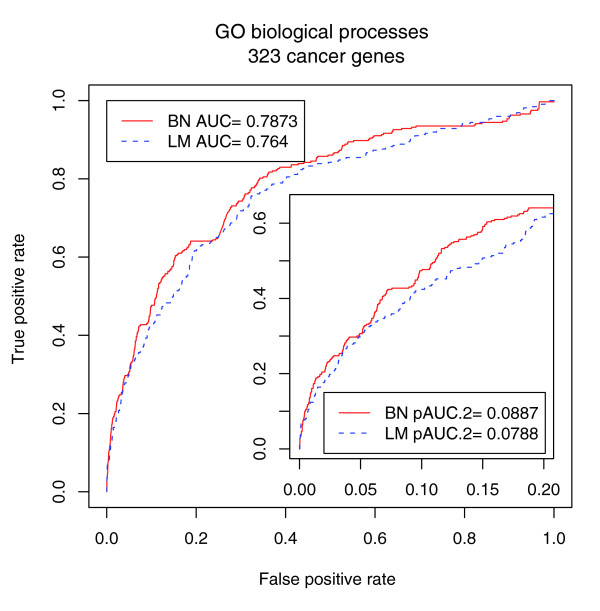
**The ROC curve, AUC and pAUC.2 of 100-fold cross-validation in predicting cancer genes using GO BPs**. BN represents the Bayesian network model, and LM represents the local Bayesian model.

Third, the CAN-processes inferred from the BN model are superior to the results obtained by the local strategy in predicting unknown cancer genes as evaluated by cross-validation. Similar to the case of lethality, we employed cross-validation to compare the BN model and the LM model in predicting cancer genes in 'cancer-gene census'. We measured both the standard AUC and pAUC.2 as before. The results shown in Figure 6 are consistent with the results for lethality. The improvement of the BN model over the LM model is more significant at a low false positive rate. The pAUC.2 increases by 12.7%, and the standard AUC increases by 3%. Compared with the case of lethality, the improvement here is smaller (pAUC.2 increases by 46.9% when GO BPs are used in the case of lethality). The reasons are that our knowledge of cancer genes is far from complete, that the proportion of cancer genes in the CAN-processes is much lower than the proportion of lethal genes in lethal complexes, and that human genes are not as well annotated as yeast genes. For example, more than 50% of human genes (more than 40% of cancer genes) are annotated only with most general GO BPs (GO BP size >100). For those genes, it is unlikely for any method to make an accurate prediction.

Last, but equally important, comparison of CAN-processes prioritized in different cancer gene datasets shows that the BN model results are more consistent with each other than the HG enrichment test results. In order to show the consistency of CAN-processes prioritized in different cancer gene datasets, a second group of cancer genes was considered. These cancer genes were identified recently through systematic sequencing of colorectal and breast cancer genomes for somatic mutations [20] and are referred to as Wood's dataset (see Materials and methods for details). The same process and cutoff were used as before to generate a list of the top CAN-processes by the BN model. These CAN-processes together with the same number of top CAN-processes ranked by the HG enrichment test are shown in Table 2. Between the 1,137 and 973 genes involved in the two sets of CAN-processes prioritized by the BN model in the two datasets, respectively, a total of 101 are common to both. The overlap is statistically significant as measured by the HG *p*-value for overrepresentation at 0.002. On the contrary, when the HG enrichment test was used, genes involved in the CAN-processes prioritized in Wood's data are significantly underrepresented when compared to those involved in the CAN-processes prioritized in 'cancer gene census' (HG *p*-value for underrepresentation is 1.9e-37). Therefore, the BN model results are more consistent with each other than the HG enrichment test results when different datasets are used.

**Table 2:** Top GO CAN-processes ranked by the BN model or the HG enrichment test based on cancer genes from systematic sequencing of colorectal and breast cancer genomes

GO CAN-processes prioritized by the BN model			GO CAN-processes prioritized by the HG enrichment test		
	
GO CAN-process	Total gene number	Cancer gene number	GO CAN-process	Total gene number	Cancer gene number
GO:0007016 cytoskeletal anchoring	10	3	GO:0007155 cell adhesion	689	34
GO:0030198 extracellular matrix organization and biogenesis	27	6	GO:0022610 biological adhesion	689	34
GO:0007185 transmembrane receptor protein tyrosine phosphatase signaling pathway	6	2	GO:0016043 cellular component organization and biogenesis	2,325	66
GO:0007155 cell adhesion	689	34	GO:0030198 extracellular matrix organization and biogenesis	27	6
GO:0007605 sensory perception of sound	116	9	GO:0048856 anatomical structure development	1,537	47
GO:0051318 G1 phase	18	2	GO:0007275 multicellular organismal development	1,797	52
GO:0006869 lipid transport	100	6	GO:0048731 system development	1,196	38
GO:0009112 nucleobase metabolic process	16	2	GO:0007519 striated muscle development	62	7
GO:0045661 regulation of myoblast differentiation	6	2	GO:0007605 sensory perception of sound	116	9
GO:0042593 glucose homeostasis	11	2	GO:0050954 sensory perception of mechanical stimulus	116	9
GO:0043534 blood vessel endothelial cell migration	6	2	GO:0016337 cell-cell adhesion	239	13
GO:0007183 SMAD protein complex assembly	4	2	GO:0032501 multicellular organismal process	3,128	73
GO:0060070 Wnt receptor signaling pathway through beta-catenin	5	2	GO:0007167 enzyme linked receptor protein signaling pathway	245	13
Median number	11	2	Median number	689	34

Although statistically significant, the overlap between the two sets of CAN-processes prioritized based on the two cancer gene datasets by the BN model is only 5% (intersection/union of genes). Since the two datasets of cancer genes differ in many respects, such a small overlap could reflect the different focus of the two datasets. Particularly, since the 'cancer-gene census' is assembled from previous studies and the Wood's dataset is derived from a recent study with new techniques, the small overlap could indicate the discovery of potentially 'new' CAN-processes by the Wood's study. To be more specific, while the majority of cancer genes in 'cancer-gene census' were detected from liquid tumors such as leukemias and lymphomas, those in Wood's dataset were identified from colorectal and breast tumors, which are normally solid tumors. In solid tumors, precursor cells must become mobile and invasive in order to become malignant. Consequently, some 'new' CAN-processes might be 'overlooked' because their disruption is not required in liquid tumors since their precursor cells are already mobile and invasive [18]. Alternatively, but not exclusively, some CAN-processes might be newly discovered as a consequence of the advantages of the systematic sequencing strategy of cancer genomes, which could be more unbiased and comprehensive than traditional cloning techniques. It is also possible that some 'new' CAN-processes were prioritized simply because of the incomplete annotation of GO, and those cancer genes involved in the 'new' CAN-processes might later be found to function in some 'old' CAN-processes.

Cytoskeletal anchoring and lipid transport are two examples of such potentially 'new' CAN-processes prioritized in the Wood's dataset by the BN model. None of the genes associated with the two GO BPs has been documented as cancer genes in 'cancer gene census'. Their potential roles in tumorigenesis or metastasis are discussed below. These findings might give insight into further study and treatment of human cancers.

#### Cytoskeletal anchoring

The involvement of cytoskeletal anchoring in cancer development is not unexpected, especially considering that it functions as a direct or indirect connection between two groups of cancer-related molecules, for example, transmembrane or membrane-associated proteins and cytoskeletal filaments, both of which are actively involved in signaling transduction, cell-cell adhesion, and other cancer-related biological processes. For example, *FLNB *and *TLN1 *are two cytoskeletal anchoring genes detected in Wood's dataset. Both of them have been detected to interact with integrins [34], a family of transmembrane receptor proteins whose key roles in tumor growth and metastasis have been explored over a long history [35]. Thus, it is speculated that malfunction of FLNB or TLN1 could contribute to cancer development by disrupting or improperly activating the functions of integrins or integrin-related signaling pathways. A similar example is SHANK1; SHANK1 has been observed to interact with Somatostatin receptor 2 [36], which was shown to be able to sensitize human cancer cells to death by ligand-induced apoptosis [37].

#### Lipid transport

Compared to cytoskeletal anchoring, the roles of lipid transport in cancers are more complicated. On the one hand, in rapidly dividing cancer cells, the availability of cholesterol is essential for proliferation and progression of the cancer [38]. On the other hand, lipid transport might directly or indirectly coordinate with many signaling pathways that control cell birth and death. For example, given that both high-density and low-density lipoprotein receptors (or receptor-related proteins) were found to regulate proliferation or survival of cancer cells [39,40], it is not surprising to find HDLBP and SORL1 in Wood's dataset. The former is a high-density lipoprotein binding protein, and the latter has been detected to interact with a low-density lipoprotein receptor-related protein-associated protein 1 (LRPAP1) [41]. Moreover, a number of lipid transporters like ATP-binding cassette (ABC) transporters have been implicated in tumor cell resistance to anticancer therapy [42]. ABCA1 in Wood's dataset might be one such example [43].

## Conclusion

In this paper, we attempt to decode phenotypic effects caused by genetic perturbation through known functional modules. By decoding gene lethality through protein complexes and investigating the conservation of gene lethality across different species in the context of lethal and non-lethal protein complexes, we provide evolutionary evidence supporting the modular nature of lethality. Based on human cancer genes, we prioritized many biological processes causally implicated in cancers, which are consistent with previous knowledge. We also identified some 'new' biological processes whose roles in cancer development are less well understood: cytoskeletal anchoring and lipid transport.

Motivated by the overlapping structure of functional modules in biological systems, we provide a global strategy to distinguish functional modules that are most likely to be actual causal modules from a large number of other 'overlapping modules' whose only relatedness with the phenotypes most likely results from the sharing of gene members with the causal modules. Local strategies, such as the HG enrichment test, ignores overlapping structures among modules and is thus less effective in distinguishing actual causal modules from the 'overlapping modules'. In contrast, the BN model filters out 'overlapping modules,' which generates a more accurate list of causal modules. Compared to either the HG enrichment test, or the LM model, in the case of prediction of gene lethality, the results consistently show that the modules prioritized by the BN model are better representatives of the actual causal modules, even though it can never be ascertained whether or not the modules prioritized by the global strategy are, indeed, true causal modules in the absence of any direct biological benchmark.

In summary, our results indicate that modularity, which is believed by investigators to be a true property of biological systems, can be applied to the interpretation of phenotypic variations from perturbations in genetic variation. This might shed light on the study of more complicated phenotypes, such as human disease. With proper modeling, the global strategy could potentially be applied to a variety of fields. For example, it might be interesting to see how it helps identify differentially expressed gene sets in microarray data analysis. More importantly, a module-based prediction strategy will benefit the study of human diseases by transferring phenotypic data learned from other organisms to human beings. This has significant implications for the treatment of human cancers.

## Materials and methods

### Bayesian network model

**Figure 7:**
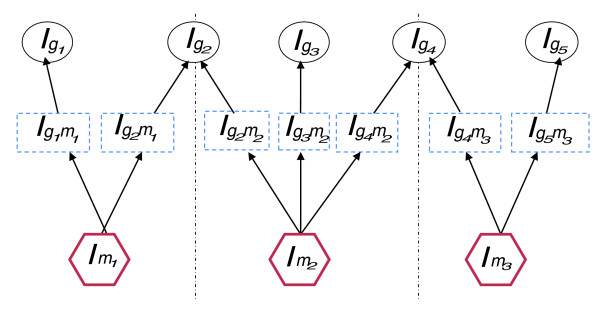
**An example of the Bayesian network**. In this network, lethality of deletion of gene *g*_*i*_, denoted as $l_{g_{i}}$, is determined by lethality of the complex-specific inactivation of its protein products, denoted as $l_{g_{i}m_{j}}$, which in turn is determined by lethality of inactivation of protein complex *m*_*j*_, denoted as $l_{m_{j}}$.

In this section, we explain the BN model we used to prioritize functional modules mediating genetic variation and its phenotypic effects. For illustration, we take single gene deletion, lethality and protein complexes as examples of the genetic perturbation, the phenotypic effect and the candidate functional modules, respectively. Figure 7 gives an example of the Bayesian network and the details are as follow.

*G*: a set of genes whose lethality is known; in Figure 7,

*G *= {*g*_1_, *g*_2 _... *g*_5_}: a set of known protein complexes; in Figure 7,

*M *= {*m*_1_, *m*_2_, *m*_3_}.

*A*_*GM *_= {$A_{g_{i}m_{j}}$|*g*_*i *_∈ *G*, *m*_*j *_∈ *M*}: is the association matrix between genes and complexes.

$A_{g_{i}m_{j}}$ = 1 if protein complex *m*_*j *_contains protein product of gene *g*_*i*_, and 0 otherwise. In Figure 7, $A_{GM}=\left(\begin{array}{ccc} 1 & 0 & 0\\ 1 & 1 & 0\\ 0 & 1 & 0\\ 0 & 1 & 1\\ 0 & 0 & 1 \end{array}\right)$.

*L*_*G *_= {$l_{g_{i}}$|*g*_*i *_∈ *G*}: is the lethality of each gene that is observed from genome-wide knockout experiments. $l_{g_{i}}$ = 1 if deletion of gene *g*_*i *_leads to inviable phenotype and 0 otherwise. In Figure 7, $L_{G}=\left\{l_{g_{1}},l_{g_{2}}...l_{g_{5}}\right\}$.

*L*_*M *_= {$l_{m_{j}}$|*m*_*j *_∈ *M*}: is the unknown lethality of each protein complex. $l_{m_{j}}$ = 1 if inactivation of protein complex *m*_*j *_leads to inviable phenotype, and 0 otherwise. We refer to those protein complexes with $l_{m_{j}}$ = 1 as lethal protein complexes, which are the causal protein complexes mediating the gene knockout and observed inviable phenotype. In Figure 7, $L_{M}=\left\{l_{M_{1}},l_{M_{2}},l_{M_{3}}\right\}$. According to the module-based explanation of lethality, the lethality observed in gene deletion is determined by the lethality of the associated protein complex(es); thus, as shown in Figure 7, there is an edge pointing from $l_{g_{i}}$ and $l_{m_{j}}$, where $A_{g_{i}m_{j}}$ = 1.

As discussed in the introduction, inference of *L*_*M *_from the observed *L*_*G *_becomes difficult when the protein products of gene *g*_*i *_participate in more than one protein complex. For example, in Figure 1, protein products of *g*_2 _participate in both protein complexes *m*_1 _and *m*_2_. If $l_{g_{2}}$ = 1, it is possible that $l_{M_{1}}$ = 1 or $l_{M_{2}}$ = 1 or both. In principle, such a problem could be solved by carrying out complex-specific protein inactivation experiments, that is, by designing a complex-specific antibody that 'knocks down' protein products of gene *g*_2 _in protein complex *m*_1 _while keeping protein products of gene *g*_2 _in protein complex *m*_2 _unchanged, and vice versa. The inviable or viable phenotypes observed in such complex-specific protein inactivation experiments could reflect the lethality of protein complexes more directly. Motivated by this, we introduce a set of hidden variables that denote the outcome of complex-specific protein inactivation experiments and function as bridges between *L*_*M *_and *L*_*G*_.

$L_{GM}=\left\{l_{g_{i}m_{j}}|g_{i}\in G,m_{j}\in M,A_{g_{i}m_{j}}=1\right\}$: are the hidden lethality data for each complex-specific protein inactivation experiment. $l_{g_{i}m_{j}}$ = 1 if inactivation of protein products of gene *g*_*i *_that participates in protein complex *m*_*j *_(while keeping other protein complexes active) leads to inviable phenotype, and 0 otherwise. In Figure 7, $L_{GM}=\left\{l_{g_{1}m_{1}},l_{g_{2}m_{1}},l_{g_{2}m_{2}},l_{g_{3}m_{2}},l_{g_{4}m_{2}},l_{g_{4}m_{3}},l_{g_{5}m_{3}}\right\}$.

The following explains the causality relationship among the three sets of variables and derives the conditional probability tables for the Bayesian network.

In reality, when gene *g*_*i *_is knocked out from the genome, all of its protein products are knocked out and hence inactivated. Thus, obviously, deletion of gene *g*_*i *_will lead to an inviable phenotype if one complex-specific inactivation of its protein products leads to an inviable phenotype. However, even when no such complex-specific inactivation leads to an inviable phenotype, there is still a possibility that the deletion of gene *g*_*i *_will lead to an inviable phenotype. One possibility is that there are agonistic interactions among protein products of gene *g*_*i *_that participate in different protein complexes such that simultaneous inactivation of them, which is the case in deletion of gene *g*_*i*_, leads to an inviable phenotype. Other possible explanations may include protein products of gene *g*_*i *_possibly acting in certain lethal modules that are not included in our dataset of candidate modules. For simplicity, we refer to all those other events or effects that cause an inviable phenotype, besides those reflected by complex-specific protein inactivation, as 'other effects' in our paper and assign them probability *P*_*other*_. According to the above arguments, the conditional probability table for each $l_{g_{i}}$ ∈ *L*_*G *_can be derived as follows:

(1) $$P\left(l_{g_{i}}=1|\left\{l_{g_{i}m_{j}}|m_{j}\in M,A_{g_{i}m_{j}}=1\right\}\right)=\{\begin{array}{ll} 1 & if\;\sum_{m_{j}\in M,A_{g_{i}m_{j}}=1}l_{g_{i}m_{j}}\geq 1\\ P_{other} & otherwise \end{array}$$

Not all members in a complex are equally important. Although individual inactivation of some members of a protein complex will cause that protein complex to 'break down' or lose its function, individual inactivation of others might not. Functional redundancy within protein complexes might be one of many possible reasons. Thus, even for a lethal protein complex, inactivation of certain members might not lead to an inviable phenotype. To accommodate such phenomena, we assign a lethal probability to each protein complex *m*_*j*_, denoted as $P_{lethal}^{m_{j}}$, representing the probability that individual inactivation of its members will lead to an inviable phenotype. Needless to say, the lethality probability will be zero for nonessential protein complexes. Thus, conditional probability tables for each $l_{g_{i}m_{j}}$ ∈ *l*_*GM *_can be expressed as:

(2) $$P\left(l_{g_{i}m_{j}}=1|l_{m_{j}}\right)=\{\begin{array}{ll} P_{lethal}^{m_{j}} & l_{m_{j}}=1\\ 0 & l_{m_{j}}=0 \end{array}$$

### Inference of module lethality given gene lethality

Our goal is to infer each variable in *L*_*M *_in the above Bayesian network, given the observed values in *L*_*G *_and the known network structure. Since there are other unknown variables *L*_*GM *_and unknown parameters $\left\{P_{lethal}^{m_{j}}|m_{j}\in M\right\}$ (the parameter *P*_*other *_is set to be a fixed value for simplicity), we employed an expectation-maximization strategy with details given below.

Initiate each $\hat{P_{lethal}^{m_{j}}}={\hat{P_{lethal}^{m_{j}}}}^{\left(0\right)}$*m*_*j *_∈ *M*

E-step: estimate each variable in the set of *L*_*GM *_and *L*_*M *_by Gibbs sampling [44], denoted as

$${\hat{L_{GM}}}^{\left(k\right)}=\left\{{\hat{l_{g_{i}m_{j}}}}^{\left(k\right)}|g_{i}\in G,m_{j}\in M\right\}\text{ and }{\hat{L_{M}}}^{\left(k\right)}=\left\{{\hat{l_{m_{j}}}}^{\left(k\right)}|m_{j}\in M\right\}$$

M-step: calculate the maximum likelihood estimation of each parameter $P_{lethal}^{m_{j}}$ as the following:

$${\hat{P_{lethal}^{m_{j}}}}^{\left(k\right)}=\sum_{gi}A_{g_{i}m_{j}}{\hat{l_{g_{i}m_{j}}}}^{\left(k\right)}/\sum_{gi}A_{g_{i}m_{j}}{\hat{l_{m_{j}}}}^{\left(k\right)}$$

### Prediction of gene lethality through cross-validation

In the prediction of gene lethality through cross-validation, we randomly chose part of the gene lethality data (training data) as known to estimate the parameters $\left\{P_{lethal}^{m_{j}}|m_{j}\in M\right\}$ of the BN model and to infer the probability of being lethal for each module $\left\{P(l_{m_{j}}=1)|m_{j}\in M\right\}$ by the above expectation-maximization strategy. The estimation results were used to infer the probability of being lethal for the remaining genes (testing data) by the following formula:

(3) $$P\left(l_{g_{i}}=1\right)=1-\prod_{m_{j}\in M,A_{g_{i}m_{j}}=1}\left(1-P_{lethal}^{m_{j}}P(l_{m_{j}}=1)\right)$$

### Local Bayesian model

As a comparison to the BN model, we also employed the LM model to infer the module lethality given gene lethality. The LM model differs from the BN model only in that only the subnetwork for a candidate module is considered as if none of its components participate in other modules (shown by dashed lines in Figure 7). Thus, formula 1 in the BN model has been simplified to the following:

(4) $$P\left(l_{g_{i}}=1|l_{g_{i}m_{j}},m_{j}\in M,A_{g_{i}m_{j}}=1\right)=\{\begin{array}{ll} 1 & if\;l_{g_{i}m_{j}}=1\\ P_{other} & otherwise \end{array}$$

Formula 2 remains the same for the LM model.

### Data sources

We first applied our BN model to the gene lethality data in *S. cerevisiae*. These data were obtained from a genome-wide gene deletion study [2], where, out of a total of 5,916 genes deleted, 18.7% (1,105) are essential for growth on rich glucose medium. In order to analyze the conservation mode of gene lethality across different species, the lethality data in *C. elegans *and *D. melanogaster *were obtained from two genome-wide RNA interference experiments [4,21]. The ortholog mapping data were downloaded from the Inparanoid database [45].

Protein complexes were treated as one type of functional module in the case of gene lethality. Both curated and HTP protein complex data were used in our analysis. The manually curated protein complex dataset was downloaded from the ScISIC dataset in the Bioconductor [46] R package *ScISI *(version 1.10.0). A detailed description can be found in [47]. It consists of protein complexes derived from small scale experiments that have been curated by GO or MIPS, and other manually curated protein complexes obtained from IntAct [48]. In total, the dataset consists of 390 protein complexes, including 582 lethal genes and 794 non-lethal genes. The high throughput (HTP) protein complex data were obtained from a recent large-scale AP-MS (affinity purification followed by mass spectrometry) experiment [49]. It consists of 491 protein complexes, including 577 lethal genes and 828 non-lethal genes.

For more broadly defined functional modules, we used GO BPs [16]. The data were downloaded from the YEASTGO dataset in the Bioconductor [46] annotation package *YEAST *(version 2.0.1) and processed with the package *GOstats *[27]. It was further processed such that, if one gene belonged to a GO node, it would be included in all of its ancestor nodes. In total, the dataset contains 2,200 GO BPs, including 1,031 lethal genes and 4,223 non-lethal genes.

We then applied our strategy to prioritize biological processes causally implicated in human cancers. We downloaded and retrieved GO annotations for human genes from the NCBI website [50]. The data were further processed in a manner similar to that for *S. cerevisiae*. In total, there are 14,371 human genes involved in 4,644 biological processes. Two datasets of cancer genes were considered. The first dataset was downloaded from the 'cancer-gene census' database, a curated cancer gene database assembled from previous studies [19]. The second dataset was obtained from systematic sequencing of colorectal and breast cancer genomes for somatic mutations [20]. In this dataset, the somatic mutations found in cancers were classified into either 'drivers' or 'passengers' [51] according to authors' criteria. Driver mutations are causally involved in the neoplastic process and are positively selected during tumorigenesis. Passenger mutations provide no positive or negative selective advantage to the tumor, but they are retained by chance during repeated rounds of cell division and clonal expansion. In the second dataset, only candidate cancer genes that are most likely to be drivers according to authors' criteria [20] are considered in our analysis. After mapping to the GO BPs, there are a total of 331 and 225 cancer genes in the two datasets, respectively.

## Abbreviations

AUC: area under the ROC curve; BN: Bayesian network; BP: biological process; CAN-processes: biological processes causally implicated in cancers; GG-NER: global genome NER; GO: Gene Ontology; HG: hypergeometric; LGLC: lethal gene whose protein product has been involved in certain lethal protein complexes; LGNLC: lethal gene whose protein product has not been involved in any lethal protein complexes; LM: local Bayesian model; NER: nuclear excision repair; NLGLC: non-lethal gene whose protein product has been involved in certain lethal protein complexes; NLGNLC: non-lethal gene whose protein product has not been involved in any lethal protein complexes; pAUC: partial AUC; ROC: receiver operating characteristic; TC-NER: transcription-coupled NER.

## Authors' contributions

TC, FS and LW conceived of and designed the study. LW carried out all the analysis and wrote the manuscript. All authors viewed and approved the manuscript.

## Additional data files

The following additional data are available with the online version of this paper. Additional data file 1 provides a description of the simulation study, Table S1, and Figures S1 and S2. Additional data file 2 is a figure plotting the 27 GO CAN-processes prioritized by the BN model (yellow) and their offspring and ancestor nodes (blue). The nodes with red circles represent 23 out of 27 GO CAN-processes prioritized by the HG enrichment test. The size of the nodes is proportional to the minus log *p*-value of the HG enrichment test for the cancer genes. Those nodes with size zero are insignificant nodes by HG enrichment test (P-value > 0.05).

## Supplementary Material

Additional data file 1 Table S1: 94 curated lethal protein complexes identified by the BN model. The complex ID starting with 'GO', 'MIPS' and 'EBI' represents GO, MIPS and Intact ID, respectively. Total # denotes the total number of genes whose lethality is known. Lethal # denotes the number of genes that are lethal. Figure S1: genes in *S. cerevisiae *are classified into four groups according to their lethality and the lethality of protein complexes to which they belong. Within each group, the pie chart represents the distribution of genes with respect to the lethality of their orthologs in *D. melanogaster*. The lethal protein complexes were identified as in Figure 1b. Figure S2: simulation results. The performance of the BN model and the HG method in identifying lethal complexes given different degrees of overlap among protein complexes and different distributions of the proportion of lethal genes in a lethal complex. Click here for file

Additional data file 2 The 27 GO CAN-processes prioritized by the BN model (yellow) and their offspring and ancestor nodes (blue). The nodes with red circles represent 23 out of 27 GO CAN-processes prioritized by the HG enrichment test. The size of the nodes is proportional to the minus log *p*-value of the HG enrichment test for the cancer genes. Those nodes with size zero are insignificant nodes by the HG enrichment test (*p*-value > 0.05). Click here for file
