# Supplementary material for: Prioritizing functional modules mediating genetic perturbations and their phenotypic effects: a global strategy
Source: Genome Biol. 2008 Dec 16;9(12):R174. doi: 10.1186/gb-2008-9-12-r174 (PMC2646278; doi:10.1186/gb-2008-9-12-r174)

Simulation study

The purpose of this simulation study is to quantify the sensitivity of the BN and HG method to two factors which could affect their performance in identifying causal modules among overlapping sets. In the context of lethal protein complexes prediction, the two factors considered in the study are (1) the degree of overlap among protein complexes, (2) the proportion of lethal genes in a lethal complex.

The parameters we used to simulate protein complexes, complex lethality and gene lethality are from the 390 curated protein complexes or the 94 curated lethal protein complexes identified by the BN model. Below are details about the simulation study.

(a) *Simulate protein complexes.* From a pool of 1376 genes (the total number of genes associated with the 390 curated protein complexes), we simulate n protein complexes. Each simulated protein complex is a random sample of the 1376 genes. The complex size follows the distribution of that of the 390 curated protein complexes.

(b) *Simulate protein complex lethality.* We randomly assign 24% of the n protein complexes simulated in (a) as lethal protein complexes, and the rest as nonlethal protein complexes. 24% is the proportion of lethal protein complexes (94 out of 390) among the curated protein complexes identified by the BN model.

(c) *Simulate gene lethality*. The lethality of each gene is set to nonlethal at the beginning. For each lethal protein complex simulated in (b), we randomly sampled a proportion p of its members, and set the lethality of their corresponding genes as lethal. p follows the distribution of P, where

P denotes the proportion of lethal genes among the 94 curated lethal protein complexes identified by the BN model.

In order to test the algorithms' sensitivity to different degrees of overlap, we altered the number of simulated protein complexes n in (a) from 100 to 1000 with an interval of 100. As n increases, we would expect higher overlap among the simulated protein complexes. We further quantified the degree of overlap by measuring the proportion of genes that are involved in more than one complex. In order to test the algorithms' sensitivity to different proportions of lethal genes in a lethal protein complex, we altered the distribution of p from the distribution of P to that of 1/2 P and 1/4 P.For each given n and given distribution of p, we repeated step (a) to (c) for 100 times to obtain 100 simulated datasets.

For each simulated dataset, we applied the BN method to infer the complex lethality given the gene lethality and complex membership simulated above. We measured the prediction accuracy by both the standard AUC and pAUC.2 as introduced in the manuscript. As a comparison, we also applied the HG test to the simulated dataset. Figure S2 gives the box plot of AUC and pAUC.2 for both methods under different degrees of overlap and different distributions of p.

As shown in Figure S2, over the range of the two factors tested here, the BN model consistently performs better than the HG method. It can be seen that as the degree of overlap increases or the proportion of lethal genes in a lethal complex decreases, the performance of both methods decrease. The amount of improvement of the BN model over the HG method also varies with the two factors. When the proportion of lethal genes in a lethal complex is relatively high, for example, when the distribution of p equals to that of P , the amount of improvement of the BN model over the HG method increases steadily as the degree of overlap increases. But when the

proportion of lethal genes in a lethal complex is relatively low, the amount of improvement of the BN model over the HG method remains relatively small for the whole range of degree of overlap tested here. The amount of improvement even starts to decrease slightly as the degree of overlap further increases.

Table S1: 94 curated lethal protein complexes identified by the BN model. The complex ID starting with "GO", "MIPS" and "EBI" represents GO, MIPS and Intact ID, respectively. Total # denotes the total number of genes whose lethality is known. Lethal # denotes the number of genes that are lethal.

Complex ID	Description	Total #	Lethal #
GO:0031261	DNA replication preinitiation complex	21	21
GO:0005666	DNA-directed RNA polymerase III complex	17	17
GO:0005656	pre-replicative complex	15	15
GO:0000172	ribonuclease MRP complex	10	10
GO:0005849	mRNA cleavage factor complex	9	9
GO:0000177	cytoplasmic exosome (RNase complex)	9	9
GO:0005675	holo TFIIH complex	9	9
GO:0005655	nucleolar ribonuclease P complex	9	9
MIPS-130	Chaperonine containing T-complex TRiC (TCP RING Complex)	8	8
GO:0043614	multi-eIF complex	8	8
GO:0000145	exocyst	8	8
GO:0030915	Smc5-Smc6 complex	7	7
GO:0019774	proteasome core complex, beta-subunit complex (sensu Eukaryota)	7	7
GO:0042729	DASH complex	7	7
EBI-1250459		6	6
GO:0000127	transcription factor TFIIIC complex	6	6
GO:0000506	glycosylphosphatidylinositol-N-acetylglucosaminyltransferase (GPI-GnT) complex	5	5
GO:0000799	nuclear condensin complex	5	5
GO:0042765	GPI-anchor transamidase complex	5	5
EBI-1208823		5	5
GO:0000120	RNA polymerase I transcription factor complex	5	5
MIPS-445.10	SCF-CDC4 complex	5	5
GO:0000214	tRNA-intron endonuclease complex	4	4
GO:0000818	nuclear MIS12/MIND type complex	4	4
MIPS-290.20.10	Tim22p-complex	4	4
EBI-1209054		4	4
MIPS-310.10	NSP1 complex	4	4
EBI-852570		4	4
GO:0031518	CBF3 complex	4	4
GO:0000928	gamma-tubulin small complex, spindle pole body	3	3
GO:0005662	DNA replication factor A complex	3	3
GO:0000126	transcription factor TFIIIB complex	3	3
MIPS-180.30	Geranylgeranyltransferase II (GGTase II)	3	3
EBI-1248655		2	2
GO:0005672	transcription factor TFIIA complex	2	2
GO:0005673	transcription factor TFIIE complex	2	2
GO:0005785	signal recognition particle receptor complex	2	2
GO:0005835	fatty acid synthase complex	2	2

GO:0005953	CAAX-protein geranylgeranyltransferase complex	2	2
GO:0009328	phenylalanine-tRNA ligase complex	2	2
GO:0017059	serine C-palmitoyltransferase complex	2	2
GO:0017087	mitochondrial processing peptidase complex	2	2
GO:0018444	translation release factor complex	2	2
GO:0031501	mannosyltransferase complex	2	2
GO:0031510	SUMO activating enzyme complex	2	2
GO:0031515	tRNA (m1A) methyltransferase complex	2	2
GO:0042272	nuclear RNA export factor complex	2	2
GO:0043541	UDP-N-acetylglucosamine transferase complex	2	2
GO:0030684	preribosome	2	2
MIPS-260.40	NSF-SNAP complex	2	2
MIPS-440.30.10.20	Prp9p/Prp11p/Prp21p complex	2	2
MIPS-500.10.110	eIF4E/eIF4G/Pab1p complex	2	2
MIPS-510.190.190	NC2 complex	2	2
GO:0032777	Piccolo NuA4 histone acetyltransferase complex	2	2
GO:0032116	cohesin loading complex	2	2
GO:0035101	FACT complex	2	2
GO:0030690	Noc1p-Noc2p complex	2	2
GO:0030691	Noc2p-Noc3p complex	2	2
MIPS-310.20	NSP1-NUP82 complex	2	2
MIPS-290.20.20	Tim17p-complex	2	2
GO:0005669	transcription factor TFIID complex	14	13
MIPS-410.35	Replication complex	20	18
GO:0005732	small nucleolar ribonucleoprotein complex	19	17
GO:0046540	U4/U6 x U5 tri-snRNP complex	25	22
GO:0008540	proteasome regulatory particle, base subcomplex (sensu Eukaryota)	8	7
GO:0005847	mRNA cleavage and polyadenylation specificity factor complex	15	13
GO:0019773	proteasome core complex, alpha-subunit complex (sensu Eukaryota)	7	6
EBI-1250245		7	6
MIPS-270.10	Inner Kinetochor Protein Complex	7	6
GO:0005665	DNA-directed RNA polymerase II, core complex	12	10
GO:0005744	mitochondrial inner membrane presequence translocase complex	6	5
GO:0008541	proteasome regulatory particle, lid subcomplex (sensu Eukaryota)	10	8
GO:0030687	nucleolar preribosome, large subunit precursor	5	4
GO:0005851	eukaryotic translation initiation factor 2B complex	5	4
GO:0032040	small subunit processome	37	29
GO:0030127	COPII vesicle coat	8	6
GO:0031105	septin complex	4	3
GO:0030869	RENT complex	4	3
GO:0005681	spliceosome	29	21

GO:0005736	DNA-directed RNA polymerase I complex	14	10
GO:0001405	presequence translocase-associated import motor	7	5
GO:0016592	Srb-mediator complex	7	5
GO:0005852	eukaryotic translation initiation factor 3 complex	7	5
GO:0030008	TRAPP complex	10	7
MIPS-60	Anaphase promoting complex (APC)	10	7
GO:0000243	commitment complex	11	7
GO:0016586	RSC complex	16	10
GO:0031932	TORC 2 complex	7	4
GO:0005742	mitochondrial outer membrane translocase complex	7	4
GO:0008250	oligosaccharyl transferase complex	9	5
MIPS-520.10	ER protein-translocation complex (Sec complex)	9	5
GO:0031011	INO80 complex	9	5
MIPS-310	Nuclear pore complex (NPC)	22	12
GO:0000783	nuclear telomere cap complex	11	5

Figure S1: Genes in *S. cerevisiae* are classified into four groups according to their lethality and the lethality of protein complexes to which they belong. Within each group, the pie chart represents the distribution of genes with respect to the lethality of their orthologs in *D. melanogaster*. The lethal protein complexes were identified as in Figure 1(b).

	**Involved in Lethal Complexes**	**Not involved in Lethal Complexes**
**Lethal Genes**	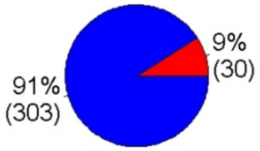	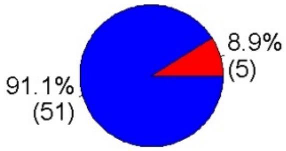
**Non-lethal Genes**	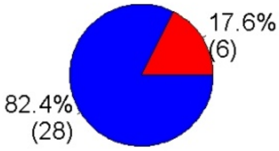	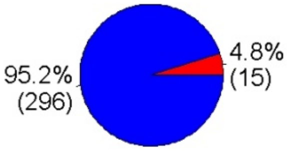

genes whose orthologs in *D. melanogaster* are related to cell growth and viability

genes whose orthologs in *D. melanogaster* are related to cell growth and viability

Figure S2: simulation results. The performance of the BN model and the HG method in identifying lethal complexes given different degrees of overlap among protein complexes and different distributions of the proportion of lethal genes in a lethal complex.

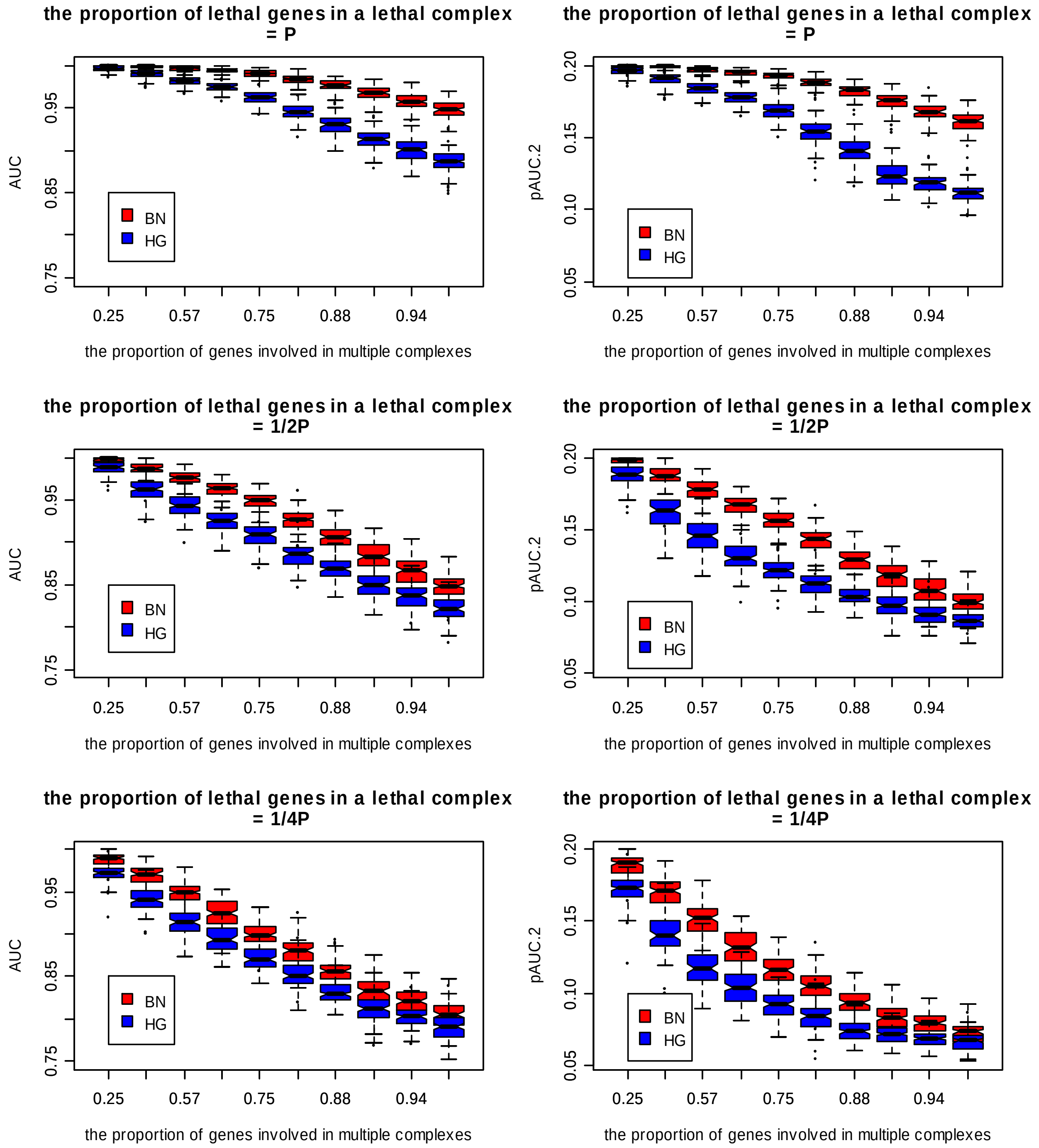

Supplement: Additional data file 1 — Table S1: 94 curated lethal protein complexes identified by the BN model. The complex ID starting with 'GO', 'MIPS' and 'EBI' represents GO, MIPS and Intact ID, respectively. Total # denotes the total number of genes whose lethality is known. Lethal # denotes the number of genes that are lethal. Figure S1: genes in S. cerevisiae are classified into four groups according to their lethality and the lethality of protein complexes to which they belong. Within each group, the pie chart represents the distribution of genes with respect to the lethality of their orthologs in D. melanogaster. The lethal protein complexes were identified as in Figure 1b. Figure S2: simulation results. The performance of the BN model and the HG method in identifying lethal complexes given different degrees of overlap among protein complexes and different distributions of the proportion of lethal genes in a lethal complex. [file gb-2008-9-12-r174-S1.pdf]
