# Supplementary figures and images for: Prioritizing functional modules mediating genetic perturbations and their phenotypic effects: a global strategy
Source: Genome Biol. 2008 Dec 16;9(12):R174. doi: 10.1186/gb-2008-9-12-r174 (PMC2646278; doi:10.1186/gb-2008-9-12-r174)

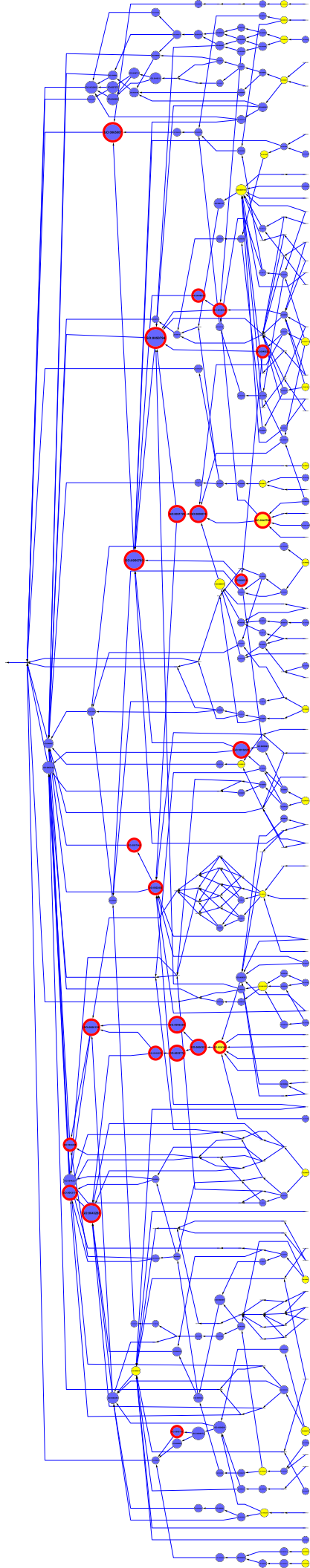

Supplement: Additional data file 2 — The 27 GO CAN-processes prioritized by the BN model (yellow) and their offspring and ancestor nodes (blue). The nodes with red circles represent 23 out of 27 GO CAN-processes prioritized by the HG enrichment test. The size of the nodes is proportional to the minus log p-value of the HG enrichment test for the cancer genes. Those nodes with size zero are insignificant nodes by the HG enrichment test (p-value > 0.05). [file gb-2008-9-12-r174-S2.pdf]
